# Development of an Electrochemical Immunosensor for Detection of Cardiac Troponin I at the Point-of-Care

**DOI:** 10.3390/bios11070210

**Published:** 2021-06-26

**Authors:** Tsung-Han Lee, Lung-Chieh Chen, Erick Wang, Chin-Cheng Wang, Yan-Ren Lin, Wen-Liang Chen

**Affiliations:** 1 Department of Emergency and Critical Care Medicine, Changhua Christian Hospital, Changhua 500, Taiwan; 169118@cch.org.tw; 2 Department of Biological Science and Technology, National Yang Ming Chiao Tung University, Hsinchu 300, Taiwan; styleme21.bt01@g2.nctu.edu.tw (L.-C.C.); erickwang51.bt05@nctu.edu.tw (E.W.); lakewang01.c@nycu.edu.tw (C.-C.W.); 3 School of Medicine, Kaoshiung Medical University, Kaoshiung 80708, Taiwan; 4 School of Medicine, Chung Shan Medical University, Taichung 40201, Taiwan

**Keywords:** cardiac troponin I, immunosensor, point-of-care

## Abstract

Cardiac troponin I (cTnI) plays an important role in the assessment of various cardiac diseases. However, accurate detection of cTnI at the point-of-care (POC) remains unfeasible. In this study, we report the development of an electrochemical immunosensor designed for rapid and accurate cTnI detection in pre-hospital settings. Rapid cTnI analysis of whole blood samples was then performed. cTnI measurements were highly correlated with the results of the standard clinical laboratory method for cTnI detection. The results of this study suggest that the proposed POC immunosensor can deliver fast and accurate cTnI analysis in pre-hospital settings to achieve rapid diagnosis and guide patient management.

## 1. Introduction

Cardiac troponin I (cTnI) is a structural protein found in the contractile apparatus of myocardial cells [1]. As it is expressed exclusively in myocardiocytes and released upon damage to cardiac tissue, cTnI is an extremely useful marker for early indication of various cardiac diseases. Plasma cTnI level is used to diagnose heart conditions such as coronary artery disease, hypertensive heart disease, and myocarditis [2,3,4]. cTnI can also serve as an early indicator of cardiovascular events such as acute heart failure or myocardial infarction (MI) that cause sudden and often severe myocardial injury [1,5], and can be combined with other test results such as electrocardiogram (ECG) reading to deliver more accurate diagnosis and improve risk stratification [5]. In the occurrence of non-ST-segment elevation myocardial infarction (NSTEMI), accurate detection of cTnI is absolutely critical to patient diagnosis and treatment, as other commonly used methods of analyzing heart abnormalities like ECG are ineffective [6]. Overall, cTnI is an indispensable biomarker that is highly useful in management of various heart problems.

Early detection of cardiac damage is imperative to patient prognosis. For example, clinical guidelines require that patients with NSTEMI must be diagnosed using plasma cTnI level and treated within 24 h to reduce the risk of further cardiac necrosis and possibility of transitioning to ST-segment elevation myocardial infarction (STEMI) [6]. Therefore, there is urgent need for a method capable of delivering immediate, accurate cTnI assessment upon patient presentation. Notably, devices designed for this purpose must be able to provide real-time detection to assist in instant diagnosis and allow for informed decision-making in a pre-hospital setting. In other words, such devices should possess various point-of-care (POC) qualities including speed, accuracy, portability, and ease-of-use. Implementation of such devices for pre-hospital cTnI analysis (i.e., in an ambulance) has been shown to significantly improve prediction of adverse heart events, especially MI [7,8], thereby reducing the time needed for patients to receive appropriate treatment [9] and improving prognosis. The focus of many recent studies has been improving the speed and accuracy of cTnI detection, which are both essential for achieving POC testing. Still, limitations—such as imprecise measurement, bulky equipment, high sample volume requirement, need for sample processing, and requisite training before operation exist—and a device that overcomes such constraints while providing fast and accurate measurement remains elusive. Therefore, true POC detection has yet to be achieved. 

The goal of this research was to develop a device capable of accurately and quantitatively detecting cTnI in POC settings. Using previously established methods [10], we constructed an electrochemical label-free biosensor modified with anti-cTnI antibodies for cTnI detection. Blood samples from cardiac damage-induced mice were tested using the developed biosensor as well as the standard clinical laboratory measurement method. Then, the biosensor was used to detect cTnI levels in clinical blood samples. Overall, results show that detection with the developed biosensor closely matches ELISA results in both mouse and human whole blood. Notably, the described sensor requires a low sample volume (20 microliters) of whole blood for rapid detection (within 5 min). In addition, the sensor is compact and portable, and the samples do not require processing. Therefore, the proposed device is highly accessible and suitable for rapid testing of cTnI to assist in the diagnosis of various cardiac diseases, and shows potential for use in POC settings such as ambulances or emergency rooms where quick and reliable screening of myocardial biomarkers is crucial for improving patient prognosis. 

## 2. Materials and Methods

### 2.1. Materials and Apparatus

Mercaptoundecanoic acid (11-MUA), N-hydroxysuccinimide (NHS), N-ethyl-N’-(3-dimethylaminopropyl) carbodiimide hydrochloride (EDC), Isopropyl β-D-1-thiogalactopyranoside (IPTG), potassium hexacyanoferrate(II) trihydrate, potassium hexacyanoferrate(III), and isoproterenol (ISO) were ordered from Sigma Aldrich, Inc., Taiwan. The pET-30a(+) vector was purchased from Novagen, Inc., Taiwan, Ni-NTA resin was ordered from BioSmart, Inc., Taiwan, and NHS-PEG_4_-biotinylation kit was purchased from Thermo Fisher Scientific, Inc., Taiwan. Anti-cTnI antibody was purchased from Proteintech, Inc., Taiwan. Recombinant Mouse cTnI protein was ordered from Abcam, Inc., Taiwan. Human cTnI native protein was purchased from MyBiosource, Inc., Taiwan. Male mice (Balb/c) were ordered from BioLASCO Taiwan Co., Taiwan. The ADVIA Centaur^®^ XPT Immunoassay System and Beckman Coulter^®^ Access Immunoassay System were utilized for standard clinical laboratory assay.

### 2.2. Biomediator Expression and Fabrication of cTnI Biosensor Chips

To produce the biomediator used for biochip functionalization, GW linker (GINSSSVPGDPPW) was constructed with a streptavidin sequence and cloned into the pET-30a(+) vector for recombinant protein expression. *Escherichia coli* (*E. coli*) DH5α was used for plasmid cloning and propagation, while *E. coli* BL21 (DE3) was used for biomediator expression. Biomediator expression was regulated by IPTG induction and purification was performed according to a standard Ni-NTA column protocol.

For biosensor chip production, we obtained gold electrodes produced using semiconductor manufacturing technology under optimized settings to ensure high purity and consistency. Then, functionalization of biochips was performed according to previously established methods [10]. Briefly, electrodes were first immersed in a 10 mM ethanolic solution of 11-MUA for 48 h to allow for self-assembled monolayer (SAM) formation. SAM was then activated with 100 mM EDC/NHS solution for 1 h. Once activated, biochips were washed, then immersed in a 10 μg/mL biomediator solution for covalent bonding for 1 h. After immobilization of biomediator, chips were washed and blocked with 3% gelatin solution for 1 h. Finally, cardiac enzyme antibodies were biotinylated using the NHS-PEG_4_-biotinylation kit, and biochips were immersed in 1 μg/mL solution of biotinylated antibodies, resulting in functionalized biochips. All reactions were carried out at room temperature. Further details regarding biomediator design and expression as well as biochip functionalization can be found in our previously published work [10].

### 2.3. Detection Procedure of the Handheld Immunosensor Device

**Figure 1:**
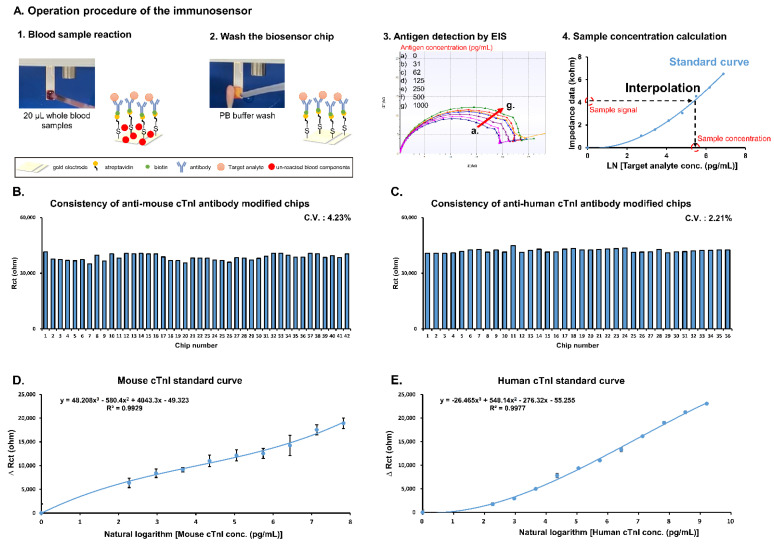
Establishment of immunosensor for cTnI detection in whole blood samples. (**A**) Detection procedure used to obtain standard curves. (**B**) Investigation of consistency of individual anti-mouse cTnI antibody modified biochips after functionalization. (**C**) Investigation of consistency of individual anti-human cTnI antibody modified biochips after functionalization. (**D**) Standard curve of mouse cTnI (analysis was performed in triplicate). (**E**) Standard curve of human cTnI (analysis was performed in triplicate).

Detection using the immunosensor occurs in three steps: first, 20 μL of whole blood mouse or patient sample is dropped on the reaction area of the biosensor chip for 3 min; secondly, the reaction area is washed by 20 mM phosphate buffer (ph 7.4) to remove the un-reacted blood components; thirdly, detection buffer (1x phosphate-buffered saline with 2 mM K_3_[Fe(CN)_6_]/K_4_[Fe(CN)_6_] (1:1) mixture) is added to the reaction surface and the detection button is pressed. Electrochemical impedance spectroscopy (EIS) was applied to analyze the biosensor chips in the frequency ranged from 0.1–1 kHz with an amplitude of 10 mV (Figure 1(A3)). The impedance data (charge transfer resistance (Rct)) was acquired using the software ZSimpWin in the simulation by the Randles’ equivalent circuit: R(Q(RW)). A standard curve was produced using the standard antigen and the formula was input into the software of the device. The impedance data of whole blood sample was interpolated into the formula and the concentration of cTnI was calculated by the software of device (Figure 1(A4)). 

### 2.4. Establishment of Cardiac Damage Mouse Model

All animal experiments were approved by the Animal Care and Use Committee of Changhua Christian Hospital (IACUC approval no. CCH-AE-108-020). Weight and heartrate of male 10-week-old Balb/c mice were recorded. Once measurements were recorded, a cardiac damage mouse model was established via subcutaneous (SC) injection of isoproterenol (ISO) in line with previously reported methods [11,12]. Mice were randomized into group A (SC injection of 0.5 mL saline once every 24 h for 2 days and blood taken 24 h after final injection), group B (SC injection of 100 mg/kg ISO in 0.5 mL saline once every 24 h for 2 days and blood taken 24 h after final injection), and group C (SC injection of 200 mg/kg ISO in 0.5 mL saline once every 24 h for 2 days and blood taken 24 h after final injection).

### 2.5. Examination of Cardiac Damage Mouse Model

Twenty-four hours after the second dose of the ISO, mice were anaesthetized using isoflurane and retro-orbital bleeding was performed to collect samples for cTnI analysis. Samples were split into two groups; one group was sent to National Chiao Tung University for handheld immunosensor analysis and the other group was immediately sent to the standard clinical laboratory. For the standard clinical laboratory assay, blood samples were preprocessed to obtain serum and the ADVIA Centaur^®^ XPT Immunoassay System was used in the Union Clinical Laboratory for ELISA analysis. After blood samples were taken, one mouse from each treatment group was randomly selected for autopsy. The heart was removed and fixed in 10% formalin solution (4% formaldehyde). Then we sent the heart samples to the Research Center for Animal Medicine in National Chung Hsing University for histopathological examination by Dr. Jiunn-Wang Liao of the Graduate Institute of Veterinary Pathobiology. 

### 2.6. cTnI Analysis of Patient Samples

From May 2020 to November 2020, blood samples from 32 patients at Changhua Christian Hospital Emergency Department whose chief complaints were chest pain and dyspnea were collected. After their conditions were stabilized, blood was drawn from the patient and cTnI analysis was subsequently performed on all samples using both the immunosensor and the standard clinical laboratory high sensitivity troponin I (Hs-cTnI) assay method. 

### 2.7. Statistical Analysis

Mixed design ANOVA was used to analyze weight, heartbeat and myocardial enzymes of mice. We list all the test values measured by the ELISA and the immunosensor, and use the graphical regression of R-squared for analysis.

## 3. Results

### 3.1. Standard Curve of cTnI Detection Using the Handheld Immunosensor

Figure 1A describes the detection procedure for producing the cTnI standard curves using the POC immunosensor. First, biosensor chip consistency was evaluated using EIS. Quality control results indicate a coefficient of variation (CV) of 4.23% and 2.21% for anti-mouse cTnI (Figure 1B) and anti-human cTnI (Figure 1C) antibody-labeled chips, respectively. Next, for cTnI standard curves, 20 μL samples of whole blood containing various concentrations of standard mouse and human cTnI were allowed to react with anti-cTnI antibody-labeled chips followed by detection using EIS. Resulting data in the form of ΔRct (ohms) was plotted against standard cTnI concentrations. Interpolation between points resulted in the mouse cTnI standard curve shown in Figure 1D as well as the human cTnI standard curve shown in Figure 1E. The limits of detection (LOD) of mouse cTnI and human cTnI using the immunosensor were 10.91 and 6.86 pg/mL, respectively.

### 3.2. Establishment of Cardiac Damage Mouse Model

**Figure 2:**
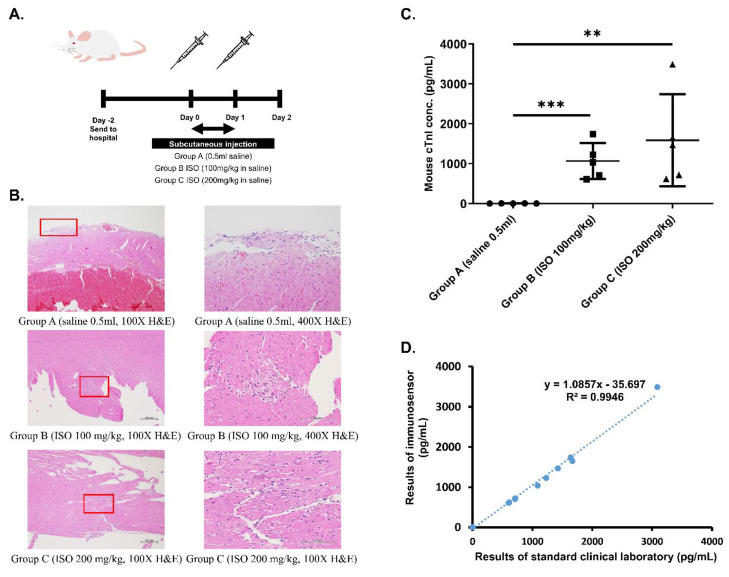
Establishment of cardiac damage mouse model. (**A**) Schedule of ISO injection in Balb/c mice. Mice were randomized into Group A (0.5 mL saline), Group B (100 mg/kg ISO in saline), and Group C (200 mg/kg ISO in saline). (**B**) IHC staining of heart tissue from mice in different groups at 100× and 400× magnification. Areas that were magnified to produce 400× images are boxed in red. (**C**) Statistical analysis of differences in cTnI measurements obtained using immunosensor between mice from the three groups. ** *p* ≤ 0.01, *** *p* ≤ 0.001. (**D**) Correlation between immunosensor detection method and standard clinical laboratory detection method in mouse model samples.

Cardiac damage mouse model was established using 10 week-old Balb/c male mice via ISO injection according to the schedule shown in Figure 2A to cause cardiac tissue damage and cTnI secretion and thus mimic patients experiencing cardiac events such as MI. 

First, physical conditions of the mice were assessed to ensure consistency of the model. Weight and heartrate of mice from the three different groups were measured before the first ISO injection (day 0) as well as before drawing blood (day 2). Prior to injection, no significant difference in both weight and heartrate existed between the three groups. After injections and before blood was drawn, mice from groups B and C exhibited significant decrease in weight as well as significant increase in heart rate following ISO injection when compared to control mice from group A (*p* < 0.05).

Next, ELISA was used to determine the level of cTnI in blood drawn from the three groups of mice. According to results shown in Table 1, control mice had normal blood cTnI concentration (<0.04 ng/dL), while ISO-injected mice exhibited significantly higher blood cTnI concentrations based on dosage (1.06 and 1.50 ng/dL for groups B and C, respectively). Finally, histopathological examination was performed to confirm the occurrence of ISO-induced myocardial necrosis. As can be seen in the hematoxylin and eosin (H&E) stain results shown in Figure 2B, myocardial damage, indicated by vacuolization and overall degradation of tissue, occurred in mice from groups B and C, but not in mice from group A. Furthermore, the severity of lesions appears to be dose-dependent, with tissue from group C exhibiting larger total inflamed area than tissue from group B.

### 3.3. Mouse cTnI Detection Results

To determine the accuracy of the proposed immunosensor, detection of cTnI in whole blood samples drawn from all mice was examined. According to immunosensor results shown in Figure 2C, mice from the three different groups exhibited statistically significant differences in blood cTnI levels, with higher ISO dosage eliciting higher cTnI levels. Immunosensor results were then compared with laboratory ELISA results using graphical linear-regression of R-squared, shown in Figure 2D. An R^2^ value of 0.9946 was obtained, indicating high correlation between the proposed method and the standard clinical laboratory method. 

### 3.4. Patients’ cTnI Detection Results

**Figure 3:**
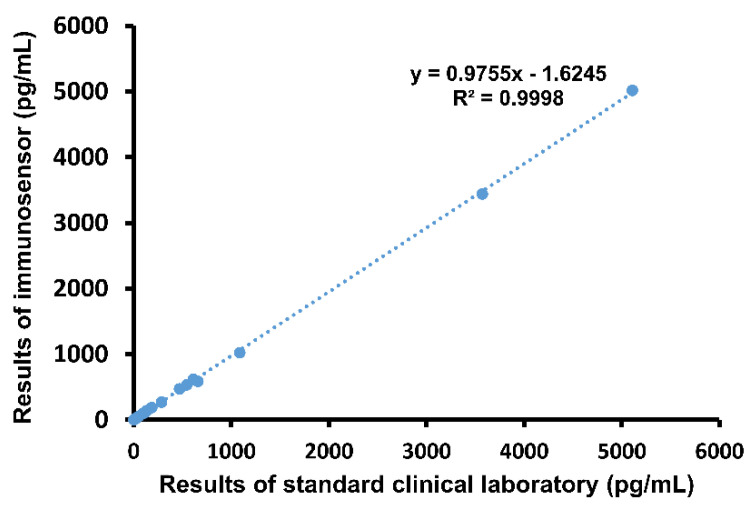
Comparison of immunosensor detection method to standard clinical laboratory detection method in human blood samples.

Next, whole blood samples drawn from patients were analyzed using the both the immunosensor and ELISA. Results were compared using graphical linear-regression of R-squared, shown in Figure 3. An R^2^ value of 0.9998 was obtained, again indicating high correlation. Notably, the proposed method of detection produced results within 5 min, while it took an average of 41.1 min for the emergency physicians to receive the same report from the hospital laboratory. 

## 4. Discussion

One of the greatest limitations in POC devices is imprecision, measured as CV, which must be as low as possible in order to achieve accurate analyte reading and improve diagnosis. Therefore, consistency of biochips used for detection was assessed with EIS. Measurement of baseline values of the chips resulted in a CV of 4.23% and 2.21% for anti-mouse cTnI (Figure 1B) and anti-human cTnI (Figure 1C) antibody-labeled chips, respectively. Importantly, these values suggest that the proposed detection method is stable enough for guideline use, falling well within the suggested CV of 10. Thus, analysis of biochip consistency indicates that the developed immunosensor provides precise detection of cTnI suitable for guiding POC diagnosis. 

Next, we confirmed the validity of the cardiac damage mouse model by measuring weight and heartrate, determining blood cTnI levels, and examining cardiac muscle for evidence of ISO-injection induced myocardial necrosis. Data shown in Table 1 indicate that, prior to injection, the three groups displayed no significant differences in weight and heartrate that would affect results of the study. Additionally, weight and heartrate values after ISO-injection suggest the occurrence of cardiac damage. Specifically, body weight decreased significantly in groups B and C after injection, a phenomenon previously proven to serve as a reliable indication of heart failure after MI in rats [13]. On the other hand, heartrate of mice in groups B and C increased significantly after injection in a dose-dependent manner. ISO, a β-adrenergic agonist and a synthetic catecholamine that produces cytotoxic free radicals [11,12], has been shown to increase heartrate while causing myocardial cell necrosis. The increased heartrate observed in group B after ISO injection as well as the further increase seen in group C verify that mice in both groups experience varying levels of heart damage according to ISO dosage, and can mimic the cardiac profile of a patient experiencing MI. After weight and heartrate were measured, blood cTnI concentration of each group was investigated by performing ELISA. Results in Table 1 indicate a significant increase in cTnI with an increase in ISO dosage, while control mice exhibited normal levels of cTnI. Finally, H&E stain results in Figure 2B clearly confirm the occurrence of myocardial cell necrosis, indicated by the greater vacuolization and degradation of cardiac tissue (boxed areas) [14]. Notably, tissue from Group C exhibited greater total inflamed area, suggesting that severity of myocardial necrosis is increased as ISO dosage increases. Overall, the observed physiological changes match those documented in previous reports of ISO-induced heart damage in mice [11,12]. Taken together, these results prove the validity of the cardiac damage mouse model.

Finally, we assessed accuracy of immunosensor detection by comparing to standard method using both blood samples from the validated cardiac damage mouse model as well as clinical blood samples. Whole blood samples drawn from the three groups of mice were analyzed using both the proposed immunosensor as well as ELISA for comparison. As shown in Figure 2D, the correlation between cTnI values obtained using the two different methods was very high, with an R-squared value of 0.9946. Then, clinical whole blood samples were analyzed in the same way. Correlation, shown in Figure 3, was also very high, with an R-squared value of 0.9998. Importantly, all immunosensor measurements were obtained within 5 min directly from whole blood samples, while cTnI analysis using the standard clinical laboratory method required sample processing and took over 40 min on average. These data indicate that the immunosensor can detect not only the presence of but also the precise concentration of cTnI in blood. Notably, blood cTnI levels have been shown to be highly correlated with severity of cardiac damage [15], suggesting that the detection results using the immunosensor can be used to guide decisions regarding treatment. Indeed, varying levels of blood cTnI in both the cardiac damage mouse model as well as patients included in this study could be accurately measured using the immunosensor. Together, the results show that the proposed immunosensor can serve as a suitable replacement for current standard clinical methods and produce near identical results using unprocessed whole blood samples to assist in diagnosis, as well as determination, of disease severity.

An immunosensor placed in an ambulance would allow medical personnel to rapidly identify the occurrence of heart damage and assist in timely management. For example, patients with NSTEMI normally must be transported to a hospital for assessment of plasma cTnI before diagnosis can be reached. Patients with severe NSTEMI may need to be relocated to a specialized facility with trained operators capable of performing operations such as percutaneous coronary intervention (PCI) or coronary artery bypass graft (CABG), causing additional delay [9]. However, rapid cTnI detection on the ambulance would allow medical personnel to determine the optimal facility based on cTnI readings. Patients with normal cTnI levels can be transported to the nearest local hospital for initial evaluation, while a patient with cTnI levels indicative of NSTEMI may be transported to a hospital with a cardiologist or that is able to perform PCI/CABG to be quickly treated before further disease progression. Notably, the reader used to analyze biochip signal in our proposed method is small (dimensions length/width/height: 21.2/8.8/6 cm) and lightweight (<2 kg). In addition, sample processing, which is normally required when using clinical laboratory standard method, is unnecessary when using the proposed biosensor. Together, these qualities would allow for highly accurate cTnI detection directly at the POC, which may help to identify cardiac damage and can streamline the process of handling patients with symptoms of cardiac damage to maximize chances of delivering appropriate care.

## 5. Conclusions

In this study, we designed a POC immunosensor capable of rapid detection of cTnI in whole blood. First, we established a cardiac damage mouse model via ISO injection to mimic the heart damage associated with cardiac events. Then, the immunosensor was used to analyze cTnI levels in whole blood samples drawn from mice, and the results were compared with results obtained using ELISA. The correlation between the detection values was very high, with an R-squared value of 0.9946. Then, clinical whole blood samples obtained from a hospital were analyzed in the same way. Again, correlation was very high, with an R-squared value of 0.9998. Overall, detection using the described immunosensor was fast and accurate. More importantly, the device is portable and easy to use, and can therefore be placed directly at the site of the patient for true POC diagnosis. In sum, the proposed immunosensor has the potential to improve efficiency identification of cardiac damage and ensure that patients receive prompt and optimal care before further disease progression.

## Figures and Tables

**Table 1:** Weight, heart rate, and cTnI measurements of the cardiac damage mouse model. All figures are expressed as Mean ± SEM, (N = 7 in control group and N = 5 in other groups), * *p* < 0.05 vs. weight before ISO injection, ^†^ *p* < 0.05 vs. heart rates before ISO injection, ^‡^ *p* < 0.05 vs. control group by standard clinical laboratory ELISA.

Group	Weight (g) (before ISO Injection)	Heart Rate (min^−1^) (before ISO Injection)	Weight (g) (48 h after ISO Injection)	Heart Rate (min^−1^) (48 h after ISO Injection)	Troponin-I (ng/dL) by ELISA (48 h after ISO Injection)
Group A (saline 0.5 mL)	26.28 ± 0.71	566.14 ± 51.32	26.24 ± 0.70	570.29 ± 37.30	0.003 ± 0.001
Group B (ISO 100 mg/kg)	26.22 ± 0.86	570.80 ± 57.76	25.54 ± 1.1 *	595.00 ± 46.27 ^†^	1.06 ± 0.42 ^‡^
Group C (ISO 200 mg/kg)	26.14 ± 0.54	568.40 ± 77.58	25.38 ± 0.63 *	591.82 ± 56.61 ^†^	1.50 ± 0.99 ^‡^

## Data Availability

Not applicable.
